# Influence of Deforestation, Logging, and Fire on Malaria in the Brazilian Amazon

**DOI:** 10.1371/journal.pone.0085725

**Published:** 2014-01-03

**Authors:** Micah B. Hahn, Ronald E. Gangnon, Christovam Barcellos, Gregory P. Asner, Jonathan A. Patz

**Affiliations:** 1 Nelson Institute, SAGE (Center for Sustainability and the Global Environment), University of Wisconsin-Madison, Madison, Wisconsin, United States of America; 2 Department of Population Health Sciences, School of Medicine and Public Health, University of Wisconsin-Madison, Madison, Wisconsin, United States of America; 3 Health Information Research Department, Oswaldo Cruz Foundation, Rio de Janeiro, Brazil; 4 Department of Global Ecology, Carnegie Institution for Science, Stanford University, Stanford, California, United States of America; Arizona State University, United States of America

## Abstract

Malaria is a significant public health threat in the Brazilian Amazon. Previous research has shown that deforestation creates breeding sites for the main malaria vector in Brazil, *Anopheles darlingi*, but the influence of selective logging, forest fires, and road construction on malaria risk has not been assessed. To understand these impacts, we constructed a negative binomial model of malaria counts at the municipality level controlling for human population and social and environmental risk factors. Both paved and unpaved roadways and fire zones in a municipality increased malaria risk. Within the timber production states where 90% of deforestation has occurred, compared with areas without selective logging, municipalities where 0–7% of the remaining forests were selectively logged had the highest malaria risk (1.72, 95% CI 1.18–2.51), and areas with higher rates of selective logging had the lowest risk (0.39, 95% CI 0.23–0.67). We show that roads, forest fires, and selective logging are previously unrecognized risk factors for malaria in the Brazilian Amazon and highlight the need for regulation and monitoring of sub-canopy forest disturbance.

## Introduction

Malaria remains a significant public health threat in Brazil despite decades of control efforts [1] and millions of dollars of financing [2]. Agriculture, timber extraction, and gold mining [3], [4] have transformed the forest fringes and created abundant larval breeding sites for *Anopheles darlingi* [4], the dominant malaria vector in Brazil [5], [6]. As a result, the transmission of “frontier malaria” has accompanied the migration of non-immune workers to the Amazon for these projects [3], [5]. In 2007, 99.9% of Brazil’s nearly 460,000 cases of malaria originated in the Amazon [1].

Driven by the expansion of cattle and soybean production [7], annual deforestation rates in the Amazon in the last decade have ranged between 6,000–28,000 km^2^/year [8]. By 2008, more than 17% of the pre-1970 forests had been cleared [8], [9]. Brazil’s National Institute for Space Research (INPE) has been monitoring deforestation in the Amazon via satellite since 1988 [8]. These data have been important for measuring the magnitude and spatial distribution of forest degradation [9], [10]. However, it has been widely recognized that these coarse estimates do not account for forest alterations that significantly thin the canopy and kill understory biomass but do not eliminate it entirely, such as selective logging and fire [7], [9]–[12]. These diffuse forest disturbances are difficult to distinguish in satellite images and are essentially invisible from space three years after logging occurs [12]. Regional estimates show that selective logging in the five timber production states overlaps with the INPE deforestation estimates by only 6% (±5%) and that logging adds 60 to 123% more forest damage than has been reported using deforestation extent alone [11].

Research linking forest alteration and malaria in the Amazon has likewise focused on the effects of deforestation. In the Peruvian Amazon, the degree of deforestation was shown to be associated with the biting rate of *A. darlingi* [13], and complementary larval studies point to an increase in preferred breeding habitat as the likely ecological mechanism [14]. On a larger scale, Olson et al. [15] found that a 4.3% increase in deforestation (from INPE’s satellite monitoring) increased malaria incidence 48% in health districts in Mancio Lima, Brazil. Studies cite an increase in larval breeding sites as the ecological link leading to an increase in *A. darlingi* (and subsequent malaria transmission), due to the species’ preference for a combination of shade and sunlight, deep, clear water with vegetation, and neutral to high pH [16]–[18], conditions generally found at forest margins rather than within an intact forest [19], [20].

As Amazonian development continues to alter the forests [7], an understanding of the impact on malaria epidemiology remains vital for targeting surveillance, treatment, and control strategies and setting land use policy that prevents surges in malaria transmission or reintroduction into areas where the disease was previously eradicated [21]. Furthermore, there is little information available on the effect of forest disturbances other than deforestation on malaria transmission and few studies that assess land use and malaria across the entire Brazilian Amazon basin. In this study, we examine deforestation, road density, fire, selective logging, and sociodemographic factors across the Brazilian Amazon from 1997–2003 and their association with malaria incidence in 2003 at the municipality (municipio) level. Our hypothesis is that our basin-wide analysis will show a similar positive association between deforestation rates and malaria incidence as seen in previous field and sub-municipality studies [13]–[15]. Further, we hypothesize that forest disturbances from roads, fire, and selective logging will further increase malaria risk in a municipality.

**Figure 1:**
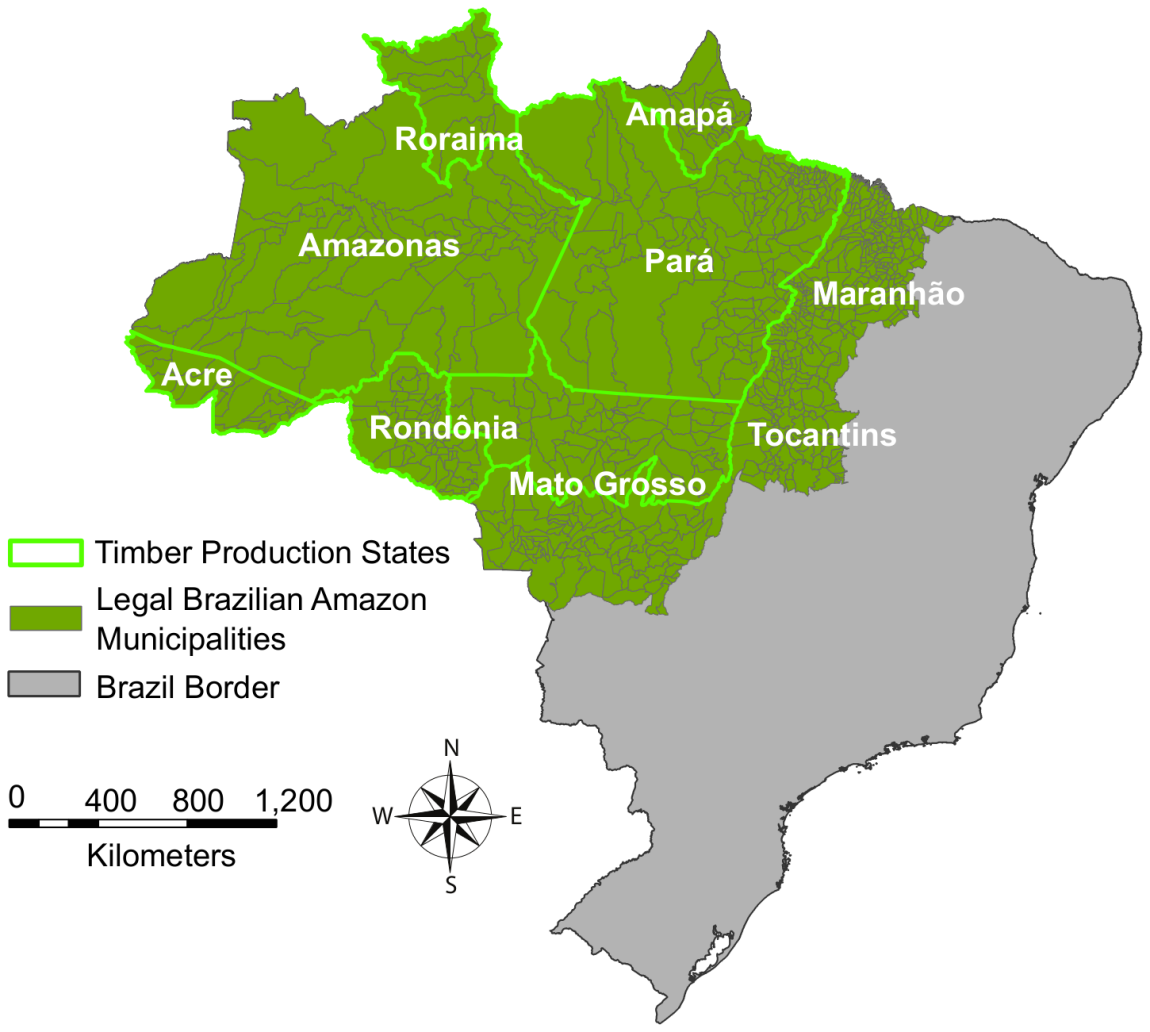
Map of the Legal Brazilian Amazon municipalities (dark green with grey borders) and the five timber production states (bright green borders): Roraima, Pará, Rondônia, Acre, and northern Mato Grosso.

Our study was conducted within the Legal Amazon of Brazil (Figure 1). The Legal Amazon region covers over half of Brazil’s land areas [7], sustains 40% of the world’s remaining tropical forests [7], [22], and hosts approximately a quarter of the world’s terrestrial species [23], [24]. The average annual temperature in the region is 22−26°C (72−79°F), and it is the wettest part of the country, with some areas receiving 2000–3000 mm of rainfall each year. The five timber production states include Roraima, Pará, Rondônia, Acre, and northern Mato Grosso (Figure 1). These states account for 90% of deforestation in the Brazilian Amazon [8], [11].

## Methods

We described human population and land cover characteristics of the Amazon municipalities and then constructed negative binomial log-linear regression models for counts of malaria cases in a municipality in 2003. For each municipality, the logarithm of the 2000 census population was included as an offset term. First, we modeled the effects of forest disturbance on malaria counts in the Legal Amazon, and then we limited our analysis to the five timber production states.

### Human Population Data and Pre-processing

Malaria incidence data by municipality in 2003 were from the System of Epidemiologic Surveillance of Malaria (SIVEP-Malaria). Operating within the Brazilian National Program for Malaria Control, the SIVEP-Malaria monitoring system is highly developed and has extensive coverage across the country due its network of public health staff who record malaria case reports via the internet [25]. Additionally, the simplicity of the collection form and free malaria medication for all cases limits data collection error and ensures high sensitivity [25]. Malaria was reported as the Annual Parasite Incidence (API), which is the number of positive blood slides per population×1000 (cases/1000 inhabitants). Malaria counts were calculated using population estimates from the 2000 census.

We controlled for several sociodemographic risk factors for malaria in the Amazon. Occupational risks were measured using a ratio of male-to-female inhabitants in the municipality and the percent of the population that worked primarily in outdoor occupations such as agriculture, forestry, or fishing and hunting. Socioeconomic status of households was measured using data from the 2000 census on the average number of people living in a household, the percent of the population who receive at or below minimum wage each month, and the percent rural and indigenous populations in each municipality. These data were also proxy measures for housing quality. Access to healthcare was measured by the average cost of transportation to the nearest state capital. GDP growth, transportation costs, and migration rates were calculated by the Brazilian Institute for Geography and Statistics (IBGE) and Brazilian Institute of Applied Economic Research (IPEA) and provided by the Oswaldo Cruz Foundation (FIOCRUZ). All other data were downloaded directly from the Brazilian census website.

### Land Cover Data and Pre-processing

We obtained data on the land cover distribution in municipalities in 2003 (percent water, remaining forest, savanna (cerrado), and deforested land) detected via Landsat imagery by INPE’s deforestation monitoring program (Programa de Cálculo do Desflorestamento da Amazônia, or PRODES). We also calculated historical deforestation rates from the PRODES data. The presence of roads has been implicated as a key driver of deforestation in the Amazon [7], and the land use change associated with road construction has been linked with malaria transmission [20]. We calculated the paved and unpaved road density in municipalities using roadway maps that were digitized by IBGE and obtained from the Woods Hole Research Center, Datasets for Amazonia and the Cerrado. We calculated the percent of the municipality affected by old (1996–99) and recent (2000–02) forest fires with data from the World Resources Institute [26]. Selective logging data were available only within the five timber production states (Figure 1). These data were derived by Asner et al. [11] using a computational analysis system (Carnegie Landsat Analysis System, or CLAS) that integrates automated image analysis, atmospheric modeling, and pattern-recognition to detect logging disturbance from Landsat ETM+imagery. Pre-processing was conducted using ENVI image analysis software (Research Systems Inc., Boulder, CO), ArcGIS 9.3 (ESRI, Redlands, CA), and Geospatial Modeling Environment [27].

### Negative Binomial Regression Modeling

All statistical analyses were performed using SAS, version 9.3 (SAS Institute Inc., Cary, NC). Sociodemographic risk factors (percent of population who migrated in the previous two years, male-to-female ratio, average number of people per household, percent rural population, percent of households living under minimum wage, average transportation costs to the nearest state capital, percent GDP growth from 2000 to 2006) and the land cover distribution in 2003 (percent of municipality that was water, remaining forest, and savanna) were included in all models as covariates. Variance inflation factors (VIFs) were used to assess multicollinearity. All VIFs were less than two in the Legal Amazon and less than three in the timber production states.

The first model in the Legal Amazon explored the following deforestation variables: absolute deforestation in 2003, percent deforestation change from 1997–2000 (rate of deforestation between 1997–2000), percent change between 2000–2001, 2001–2002, and 2002–2003. The most significant time period was retained for subsequent models. The second and third models added unpaved and paved road density (2001) and percent of the municipality affected by old fires (1996–1999) and recent fires (2000–2002), respectively. The final model assessed interactions between unpaved road density and deforestation. This stepwise modeling approach was also used for modeling the municipalities in the five timber production states. The fourth model in the timber production states included selective logging as a categorical variable – municipalities with no selective logging, municipalities where 0–7% of the remaining forest was selectively logged between 1999–2002, and municipalities where more than 7% of remaining forests were selectively logged. Although there are harvesting practices associated with “reduced impact logging” [28] there is currently no numerical threshold for extraction used to define this or other types of logging methods. Because we were assessing selective logging at the municipality level where there was no previous standard for defining logging intensity, our approach was to use graphical diagnostic plots to examine selective logging in relation to malaria incidence in order to empirically select a cut point that best described the functional relationship between logging and malaria. Coefficients are presented as risk ratios (RR), and p = 0.05 was considered statistically significant.

## Results

### Human Population and Land Cover Characteristics of Municipalities

Malaria incidence was 22.0 (±58.3) per 1000 inhabitants in the Legal Amazon and 37.7 (±76.2) per 1000 inhabitants within the timber production states (Table 1). Migration rates within the past two years were higher in the timber production states (7.0% of the population ±5.4) compared to the full Amazon (4.1% of the population ±5.5). The male-to-female ratio was just over one in all municipalities, and there were approximately 4.5 people per household throughout Amazonia. Approximately one-fifth of the population throughout the Brazilian Amazon was living at or below minimum wage in 2000. Almost half of the population made their living through agriculture, forestry, or fishing and hunting. The average cost to travel to the nearest capital in 2000 was just under US$1,000 across the Amazon, although the upper end of the range for transportation costs was lower for residents in the timber production states.

**Table 1:** Human population and land cover characteristics of municipalities.

Characteristics (year)*	N	In the LegalAmazon (n = 782)	Range	N	In the five timberproduction states(n = 289)†	Range
HUMAN POPULATION CHARACTERISTICS						
Malaria incidence per 1000 population (2003)	740	22.0±58.3	(0, 502)	282	37.7±76.2	(0, 502)
Percent of the population that has migrated in within the lasttwo years (2000)	740	4.1±5.5	(0, 41)	282	7.0±5.4	(0, 41)
Male:Female ratio (2000)	742	1.1±0.1	(0.9, 1.5)	282	1.1±0.1	(0.9, 1.5)
Average number of people living in a household (2000)	742	4.5±0.7	(3.8, 6.9)	282	4.5±0.7	(3.3, 6.9)
Percent rural population (2000)	742	47±21	(0, 98)	282	52±21	(0, 93)
Percent indigenous population (2000)	740	2.1±6.9	(0, 76)	282	2.4±8.1	(0, 74)
Percent of population who receive at or below minimumwage each month (2000)	742	23±8	(7.0, 51)	282	19±5	(7.0, 51)
Percent of population that work in agriculture, forestry orfishing/hunting (2000)	742	47±19	(1.3, 86)	282	45±18	(1.3, 81)
Transportation cost to the nearest state capitol in US$ (2000)	740	948±805	(0, 5949)	282	957±611	(14.0, 2928)
Percent GDP growth (2000–2005)	740	18±22	(-100, 91)	282	17±22	(-100, 76)
LAND COVER CHARACTERISTICS						
Percent water (2003)	634	2.7±6.1	(0, 44)	289	2.4±5.5	(0, 34)
Percent forest (2003)	634	38±34	(0, 100)	289	44±31	(0, 98)
Percent deforested land (2003)	634	35±33	(0, 100)	289	38±28	(0, 94)
Percent savanna (2003)	634	25±34	(0, 100)	289	10±19	(0, 97)
Road density (m/km^2^) (2001)	782	25±27	(0, 172)	289	31±29	(0, 172)
Percent affected by fire (1996–2002)	781	19±24	(0, 93)	289	41±31	(0, 112)
Percent logged area (1999–2002)	–	–	–	289	1.7±3.8	(0, 31)

Data presented as means ±1 SD.

Timber production states include Roraima, Pará, Rondônia, Acre, and northern Mato Grosso.

**Figure 2:**
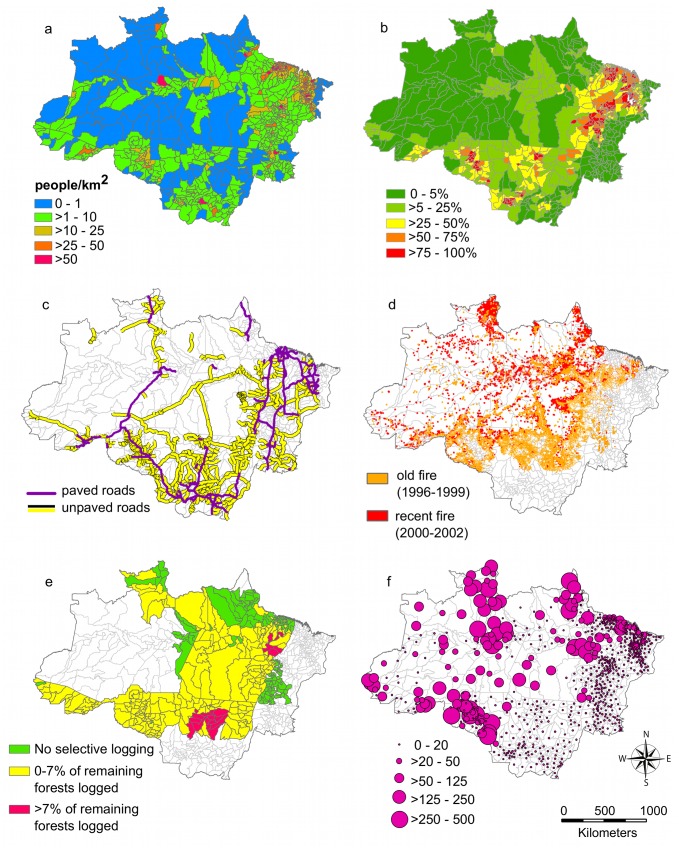
Maps of forest disturbance covariates for malaria incidence in the Brazilian Amazon: a) human population density (people/km^2^) from the Brazilian census (2000), b) percent deforestation by municipality from PRODES (2003), c) paved and unpaved roads, d) old (1996–1999) and recent (2000–2002) fires, e) percent of the remaining forest in each municipality selectively logged in the timber production states between 1999–2002, f) malaria incidence by municipality in cases per 1,000 population (2003).

In 2003, the average municipality in the Legal Amazon was 38% (±34) forested, 35% (±33) deforested, and 25% (±34) savanna (Table 1 and Figure 2). Within the timber production states, the average municipality was 44% (±31) forested, 38% (±28) deforested, and 10% (±19) savanna for the same period. The road density was 25 m/km^2^ (±27) in the Legal Amazon and 31 m/km^2^ (±29) in the timber production states. The percent of the area in a municipality affected by fire between 1996–2002 was higher within the timber production states (41%±31) compared to all of the Brazilian Amazon (19%±24). Approximately 1.7% (±3.8) of the timber production states was selectively logged between 1999–2002. This area was highly concentrated in northern Mato Grosso and northeast Pará states (Figure 2e).

### Impact of Forest Disturbance on Malaria in the Legal Amazon

We excluded municipalities where greater than 10% of the land area was unclassified due to cloud cover or where sociodemographic data were missing. Our models included 602 (77%) of the 782 municipalities in the Legal Amazon. None of the deforestation variables significantly predicted malaria rates in the first model at the broad municipality scale (Table 2).

**Table 2:** Effect of forest disturbance (deforestation, roads, and fire) on malaria incidence at the municipality level in the Legal Brazilian Amazon.

			Model 1 §			Model 2			Model 3			Model 4	
Variable*	SD†	RR‡	95% CI	p-value	RR‡	95% CI	p-value	RR‡	95% CI	p-value	RR‡	95% CI	p-value
% deforestation (1997-2000)	5.9	1.12	(0.87, 1.44)	0.37	−	−	−	−	−	−	−	−	−
% deforestation (2000-2001)	12.6	0.95	(0.80, 1.13)	0.54	−	−	−	−	−	−	−	−	−
% deforestation (2001-2002)	1.5	0.97	(0.85, 1.10)	0.63	−	−	−	−	−	−	−	−	−
% deforestation (2002-2003)	1.4	1.25	(0.97, 1.61)	0.08	1.25	(0.98, 1.60)	0.07	1.01	(0.84, 1.23)	0.88	1.05	(0.87, 1.27)	0.63
% deforested land (2003)	1.0	1.06	(0.87, 1.28)	0.58	1.08	(0.90, 1.28)	0.41	1.06	(0.89, 1.25)	0.52	1.07	(0.90, 1.27)	0.46
Unpaved road density (meters/km^2^) (2001)	19.0	−	−	−	1.51	(1.27, 1.80)	<0.0001	1.55	(1.31, 1.83)	<0.0001	0.94	(0.65, 1.36)	0.74
Paved road density (meters/km^2^) (2001)	13.4	−	−	−	1.13	(0.95, 1.36)	0.17	1.22	(1.04, 1.45)	0.02	1.18	(0.99, 1.40)	0.06
% affected by old fire (1996-1999)	18.8	−	−	−	−	−	−	1.34	(1.13, 1.57)	0.001	1.35	(1.12, 1.62)	0.001
% affected by recent fire (2000-2002)	12.4	−	−	−	−	−	−	1.37	(1.17, 1.61)	0.000	1.38	(1.17, 1.63)	0.000
Unpaved road density * % deforested land (2003)¶													
* 0-25% deforestation*	−	−	−	−	−	−	−	−	−	−	*ref*	*ref*	*ref*
* >25-50% deforestation*	−	−	−	−	−	−	−	−	−	−	1.02	(0.99, 1.05)	0.20
* >50-75% deforestation*	−	−	−	−	−	−	−	−	−	−	1.02	(0.99, 1.04)	0.16
* >75% deforestation*	−	−	−	−	−	−	−	−	−	−	1.04	(1.01, 1.06)	0.001

Variable (year of acquisition).

Residual standard deviation is the unit of change for all forest disturbance risk factors.

Risk Ratio.

Models are adjusted for several sociodemographic and environmental risk factors at the municipality level including: percent of population who migrated in the previous 2 years, male to female ratio, average number of people per household, percent rural population, percent of households living under minimum wage, average transportation costs to the nearest capitol, percent GDP growth from 2000 to 2005, and land cover in 2003 including percent of municipality that was water, remaining forest, and savanna.

Interaction between unpaved road density (meters/km^2^) and % deforestation in a municipality in 2003.

The results of our second model showed that for each additional 19.0 m/km^2^ in unpaved roads in a municipality, the malaria risk increased by 51% (p<0.0001), after controlling for human population. Paved roads did not significantly affect malaria risk when paved and unpaved road density and deforestation were the only forest disturbances included in the model.

Our third model in the Legal Amazon showed that deforestation rates were still not significant predictors of malaria incidence at the broad municipality scale, but the impact of unpaved roads increased slightly in this model, showing a 55% increase in malaria risk for each additional 19.0 m/km^2^ of unpaved roadways in a municipality (p<0.0001). Additionally, paved roads were a predictor of malaria risk, although with slightly less of an impact than unpaved roads – an additional 13.4 m/km^2^ in paved roads increased malaria risk by 22% (p = 0.02). Finally, an 18.8% increase in old fires zones or a 12.4% increase in recent fire zones increased malaria risk, 34% (p = 0.001) and 37% (p = 0.000), respectively.

Our final model in the Legal Amazon showed the impact of unpaved road density across levels of deforestation in municipalities. We found that the impact of unpaved road density on malaria risk is most prominent in municipalities with >75% deforestation.

### Impact of Forest Disturbance on Malaria within the Timber Production States

We included 246 of 289 (85%) municipalities in the timber production states with complete data in our models. The results from our first model in the timber production states showed that deforestation was not predictive of malaria risk at the municipality scale (Table 3). However, in our second model which included only recent deforestation and road density, for every 0.7% increase in deforested land in a municipality in 2003, malaria risk increased by 21% (p = 0.04). Unpaved road density was a predictor of malaria risk – for each additional 18.5 m/km^2^ of unpaved roads in a municipality, malaria risk increased 42% (p = 0.003). Paved road density did not significantly impact malaria rates in the model with deforestation and unpaved road density.

**Table 3:** Effect of forest disturbance (deforestation, roads, fire, and selective logging) on malaria incidence at the municipality level in the five timber production states (Roraima, Pará, Rondônia, Acre, and northern Mato Grosso).

		Model 1§			Model 2			Model 3			Model 4		
Variable*	SD†	RR‡	95% CI	p-value	RR‡	95% CI	p-value	RR‡	95% CI	p-value	RR‡	95% CI	p-value
% deforestation (1997–2000)	4.2	0.95	(0.73, 1.24)	0.71	–	–	–	–	–	–	–	–	–
% deforestation (2000–2001)	1.9	1.01	(0.79, 1.27)	0.96	–	–	–	–	–	–	–	–	–
% deforestation (2001–2002)	1.2	0.91	(0.74, 1.12)	0.36	–	–	–	–	–	–	–	–	–
% deforestation (2002–2003)	1.3	1.05	(0.81, 1.35)	0.72	1.07	(0.85, 1.35)	0.57	1.15	(0.86, 1.54)	0.33	1.09	(0.83, 1.43)	0.52
% deforested land (2003)	0.7	1.15	(0.97, 1.37)	0.12	1.21	(1.01, 1.44)	0.04	1.24	(1.03, 1.48)	0.02	1.22	(0.98, 1.51)	0.07
Unpaved road density(m/km^2^) (2001)	18.5	–	–	–	1.42	(1.13, 1.79)	0.003	1.40	(1.10, 1.76)	0.01	1.26	(1.00, 1.58)	0.05
Paved road density (m/km^2^) (2001)	7.9	–	–	–	1.20	(0.97, 1.48)	0.09	1.17	(0.94, 1.46)	0.15	1.21	(0.96, 1.52)	0.11
% affected by old fire(1996–1999)	17.9	–	–	–	–	–	–	0.86	(0.69, 1.07)	0.18	0.96	(0.77, 1.21)	0.75
% affected by recent fire(2000–2002)	12.2	–	–	–	–	–	–	1.15	(0.91, 1.47)	0.25	1.15	(0.92, 1.46)	0.22
0–7% remaining forestslogging¶	–	–	–	–	–	–	–	–	–	–	1.72	(1.18, 2.51)	0.005
>7–43% remainingforests logged¶,#	–	–	–	–	–	–	–	–	–	–	0.39	(0.23, 0.67)	0.001

Variable (year of acquisition).

Residual standard deviation is the unit of change for all forest disturbance risk factors.

Risk Ratio.

Models are adjusted for several sociodemographic and environmental risk factors at the municipality level including: percent of population who migrated in the previous 2 years, male to female ratio, average number of people per household, percent rural population, percent of households living under minimum wage, average transportation costs to the nearest capitol, percent GDP growth from 2000 to 2005, and land cover in 2003 including percent of municipality that was water, remaining forest, and savanna.

Logging occurred between 1999–2002.

^#^ 43% of a county’s remaining forest was the maximum amount of selective logging in a Brazilian Amazon county between 1999–2001.

In the third model in the timber production states, the impact of current deforestation on malaria increased slightly (from 21% to 24% increase in malaria risk for each additional 0.7% of land that was deforested in 2003, p = 0.02) and the impact of unpaved road density on malaria risk decreased slightly to a 40% increase in malaria risk for a 1 SD increase in unpaved road density (p = 0.01). Paved road density was still not significant, and neither recent nor old fire zones affected malaria risk.

In the final model within the timber production states, deforestation in 2003 was no longer significant. Unpaved road density was still a predictor of malaria risk, although the impact was diminished when controlling for selective logging (26% increase in malaria risk for a 1 SD increase in unpaved road density (p = 0.05)). Paved roads and forest fires were still not significant contributors to malaria risk. In comparison with areas with no selective logging, the risk for malaria was 1.72 times higher (95% CI 1.18–2.51) in areas where 0–7% of remaining forests were selectively logged between 1999–2002. In contrast, our results show the opposite association in areas where >7% of remaining forests were selectively logged, where malaria risk was 0.39 times lower than areas with no selective logging (95% CI 0.23–0.67). The most severe selective logging in our study was in Vera municipality in northern Mato Grosso where 43% of forests were affected so our results cannot be extrapolated past this level of logging impact.

## Discussion

We hypothesized that our basin-wide analysis would show a positive association between deforestation rates and malaria incidence as seen in previous field and sub-municipality studies [13]–[15]. Our study did not detect this relationship at the broad municipality level. Within the timber production states, current deforestation was a predictor of malaria when controlling for roads and fire, but was no longer significant when selective logging was considered. This inconsistent result suggests that the relationship between deforestation and malaria varies at a finer scale. Wiens [29] provides several examples, from a variety of ecological systems, of the considerable effect that the scale of analysis can have on the relationships that are uncovered. Deforestation in the Amazon is a phenomenon occurring mostly in peripheral states, such as Mato Grosso, Rondonia, Pará, north of Tocantins and west of Maranhão (Figure 2b). It also tends to be spatially clustered within municipalities [30], [31], making it difficult to match levels of deforestation and malaria incidence across municipality boundaries. Utilizing a smaller level of analysis such as the health district [15] may be the most effective scale to assess variations in malaria incidence across a deforestation gradient.

Our results confirm our second hypothesis that forest disturbances from roads, fire, and selective logging increase malaria risk in a municipality. In contrast to past measures of deforestation, these forest disturbances are more diffuse (Figure 2c–e). Consequently, municipality-level measures of roads, fire, and selective logging are likely more representative of the actual level of forest disturbance and are more appropriate to compare with municipality-level malaria data. In addition to previous research on forest disturbance and the creation of malarial mosquito breeding habitat [14], recent modeling work in the Brazilian Amazon shows that intact forest supports biodiversity of non-vector mosquito species and warm-blooded dead end hosts of malaria [32]. The former provides competition for malarial mosquitoes contending for blood meals, while the later decreases the likelihood that malarial mosquitoes will transmit the Plasmodium parasites to humans [32]. Both mechanisms suggest that forest disturbance in the Amazon could favor a rapid increase in the abundance of infected *A. darlingi* and consequently, human malaria incidence.

Although we assessed the impact of these forest disturbances on malaria independently, there is significant research demonstrating the overlap and cyclical nature of the relationships among these land use changes. In an assessment of biophysical, demographic, and infrastructural factors, Kirby et al. [7] found that unpaved and paved roads were strong predictors of deforestation in the Amazon and furthermore, in areas with no road access, there was little deforestation. Our finding that unpaved road density was most important as a predictor of malaria incidence in areas with high levels of deforestation suggests that as forest extraction activity expands around these new access points, malaria risk will likewise increase due ecological disturbance from deforestation and the road construction itself. Transportation costs are the primary constraint on logging activity in Brazil, and improved roadways would likely support expansion of this industry [33]. Similarly, it has been shown that logging increases the fire vulnerability of otherwise naturally fire resistant Amazonian forests [23], [34], and previously burned forests are more susceptible to future fire [35]. Both fire and logging load the forest floor with combustible material and open the canopy, which speeds up the rate of fuel drying [33]–[35]. An implication of these linkages is that monitoring road construction might be an immediate and practical proxy approach to identify high-risk malaria areas until malaria data are continuously available at a sub-municipality scale or deforestation can be detected at a fine enough scale across a large enough extent to monitor malaria risk associated with deforestation throughout the Amazon Basin [36].

Over half (56%) of the municipalities in the timber production states had some selective logging up to 7% of their remaining forests logged and as expected, also experienced more malaria cases compared to municipalities with no selective logging. Our finding that selective logging activity in over 7% of remaining forests in a municipality decreased malaria risk was unexpected. Holdsworth and Uhl [34] have described the significant differences in forest damage from low-impact and high-impact logging. Although these methods extract similar amounts of timber, high-impact logging creates significantly larger canopy gaps [34]. The selective logging documented by Asner et al. [11] and used for this study was dominated by high-impact logging practices that left more than half of the forest with gap fractions between 10–100% that were not fully recovered two years after harvest [37]. In these areas, it is possible that the canopy was so significantly thinned that it prevented the accumulation of water bodies on the forest floor suitable for mosquito breeding. Other studies have shown that logging has substantial impacts on the understory light environment as well as many ecological processes such as nutrient cycling and hydrological function [38]. Future research should assess rates of evapotranspiration, water accumulation, and larval breeding densities across a gradient of logging intensity. Additionally, although high-impact logging practices may initially decrease viable mosquito breeding habitat, the impact of forest regrowth on vector density is unknown. Ecosystem transformations during the recovery stages of high-impact selective logging may mirror the ecological transitions that support mosquito breeding that are generally associated with frontier malaria, such as partial shade at forest fringes [3].

The unit of analysis for this study was the municipality. On one hand, this made a basin-wide analysis feasible, but it also prevented controlling for individual level risk factors and exploring finer scale relationships between forest disturbance and malaria. Additionally, although Brazil has a highly developed malaria surveillance system, we were not able to control for differences in surveillance intensity or quality across the Amazon. The SIVEP data uses the municipality of residence for malaria cases and therefore may not capture the location of patient exposure. This may explain some of the discrepancy between deforestation rates and malaria incidence at the municipality level if a substantial number of cases are exposed while working in heavily deforested areas outside of their municipality of residence. Our analysis was not spatially explicit. If high levels of deforestation occurred in a remote area of a municipality with few inhabitants while a large urban center nearby had low levels of malaria, our aggregated municipality level results might show that high deforestation rates are associated with low malaria risk. Finally, although the inclusion of selective logging in our models represents an advance in the analysis of forest disturbance and malaria risks in the Amazon, these data may suffer from the same limitations as the binary PRODES deforestation data because they are not qualified by type of logging practice. Future studies should assess the impact of logging severity and method on malaria risk. Our results also open the door for higher resolution and spatially explicit approaches to assess the impact of forest disturbances on malaria risk in the Amazon.

In summary, we show that roads, fires, and selective logging are previously unrecognized risk factors for malaria in the Brazilian Amazon. Our approach utilized existing, high-reliability databases from government, university, and nonprofit research centers to understand the impact of forest disturbance on malaria risk in the most active land use frontier in the world [39]. As our capability to monitor forest changes via satellite continues to improve and malaria data become more readily available at finer spatial scales, these methods can provide a foundation for near real-time monitoring of disease spread to focus public health control efforts.
